# Predictive Value of Gensini Score in the Long-Term Outcomes of Patients With Coronary Artery Disease Who Underwent PCI

**DOI:** 10.3389/fcvm.2021.778615

**Published:** 2022-01-24

**Authors:** Kai-Yang Wang, Ying-Ying Zheng, Ting-Ting Wu, Yi-Tong Ma, Xiang Xie

**Affiliations:** ^1^Department of Cardiology, First Affiliated Hospital of Xinjiang Medical University, Urumqi, China; ^2^Department of Cardiology, First Affiliated Hospital of Zhengzhou University, Zhengzhou, China; ^3^Key Laboratory of Cardiac Injury and Repair of Henan Province, Zhengzhou, China

**Keywords:** Gensini score, coronary artery disease, percutaneous coronary intervention, clinical outcomes, diabetic population

## Abstract

**Objective:**

Gensini score is an effective tool used to evaluate the severity of coronary artery disease (CAD). Whether the Gensini score has predictive value for the clinical outcomes of patients with CAD after percutaneous coronary intervention (PCI) has not been investigated.

**Methods:**

All patients were from the Clinical Outcomes and Risk Factors of Patients with Coronary Heart Disease after PCI (CORFCHD-PCI), a retrospective cohort study involving 5,672 patients with CAD who underwent PCI, such as 2,110 patients with diabetes and 3,562 patients without diabetes, from January 2008 to December 2017. Patients were divided into three groups according to the tertile of Gensini score: first tertile (Gensini score <11 points), second tertile (Gensini score 11–38 points), and third tertile (Gensini score >38 points). The median follow-up time was 31.0 (interquartile range, IQR: 30.0) months. Compared the differences in clinical outcomes between the groups. Multivariate Cox regression analyses were performed to assess the predictive value of the Gensini score for outcomes over up to 10 years of follow-up.

**Results:**

In the population without diabetes, there were significant differences between the three groups in the incidences of all-cause mortality (ACM, *p* = 0.048), cardiac mortality (CM, *p* = 0.024), major adverse cardiovascular (CV) events (MACEs, *p* = 0.006), and major adverse cardiovascular and cerebrovascular events (MACCEs, *p* = 0.009). In the population with diabetes, there were significant differences between the three groups in the incidences of ACM, CM, MACEs, and MACCEs (all *p* < 0.001). After multivariate Cox regression analyses, in the population without diabetes, the respective risks of ACM, CM, MACEs, and MACCEs were increased 89.9% [hazard ratio (HR) = 1.899, 95% CI: 1.285–2.807, *p* = 0.001], 115.1% (HR = 2.151, 95% CI: 1.378–3.356, *p* = 0.001), 48.1% (HR = 1.481, 95% CI: 1.152–1.904, *p* = 0.002), and 49.8% (HR = 1.498, 95% CI: 1.176–1.907, *p* = 0.001) in the third tertile compared with those in the first tertile. In the population with diabetes, the respective risks of ACM, CM, MACEs, and MACCEs were increased 248.5% (HR = 3.485, 95% CI: 1.973–6.154, *p* < 0.001), 260.4% (HR = 3.604, 95% CI: 1.866–6.963, *p* < 0.001), 130.2% (HR = 2.302, 95% CI: 1.649–3.215, *p* < 0.001), and 119.8% (HR = 2.198, 95% CI: 1.600–3.018, *p* < 0.001) in the third tertile compared with those in the first tertile.

**Conclusion:**

The present study indicated that the Gensini score is an independent predictor of long-term adverse outcomes in patients with CAD who underwent PCI, and it has more predictive value in the population with diabetes.

## Introduction

Coronary artery disease (CAD) refers to heart disease caused by myocardial ischemia and hypoxia due to coronary artery stenosis or occlusion. It is the most common type of organ disease caused by atherosclerosis, and it is also the most common clinical cardiovascular (CV) disease (1). In recent years, with the innovation of surgical instruments and technical upgrading of percutaneous coronary intervention (PCI), more and more patients with CAD were treated with PCI, so as to obtain effective revascularization. However, in actual clinical work, it has been found that some patients have poor long-term outcomes after PCI treatment. Among patients who underwent PCI, patients with diabetes mellitus (DM) represent a high-risk subset compared with individuals without DM. Because DM is prone to have a greater atherosclerotic burden, diffuse, and long lesions in small-caliber vessels, and accelerated neointimal hyperplasia (2), patients with diabetes who underwent PCI have higher rates of adverse outcomes than patients without diabetes (3). Therefore, it is particularly important to choose a reasonable treatment strategy for CAD and identify the risk factors leading to poor outcomes. Morphology and degree of stenosis of coronary artery lesions determine the choice of the interventional treatment strategy. Currently, there are a variety of scoring systems used for quantitative analysis of coronary artery lesions and Gensini scoring is more commonly used in clinical practice. Gensini score fully considers the number, location, and degree of stenosis of coronary artery lesions, which is a more scientific evaluation standard of coronary artery lesions (4). At the same time, this scoring system has also been widely used in related studies on the clinical outcomes of CAD. At present, a number of studies have confirmed that the Gensini score can predict the risk of major adverse cardiovascular and cerebrovascular events (MACCEs) in patients with different types of CAD (5, 6) and evaluate the severity of coronary artery lesions combined with certain biochemical indicators (7–9). However, there are few studies related to the outcomes after PCI, especially the reports on the long-term outcomes. In this study, a large single-center retrospective cohort study was conducted to investigate the predictive ability of Gensini score on clinical adverse outcomes 10 years in patients with CAD who underwent PCI.

## Methods

### Study Design and Population

**Figure 1:**
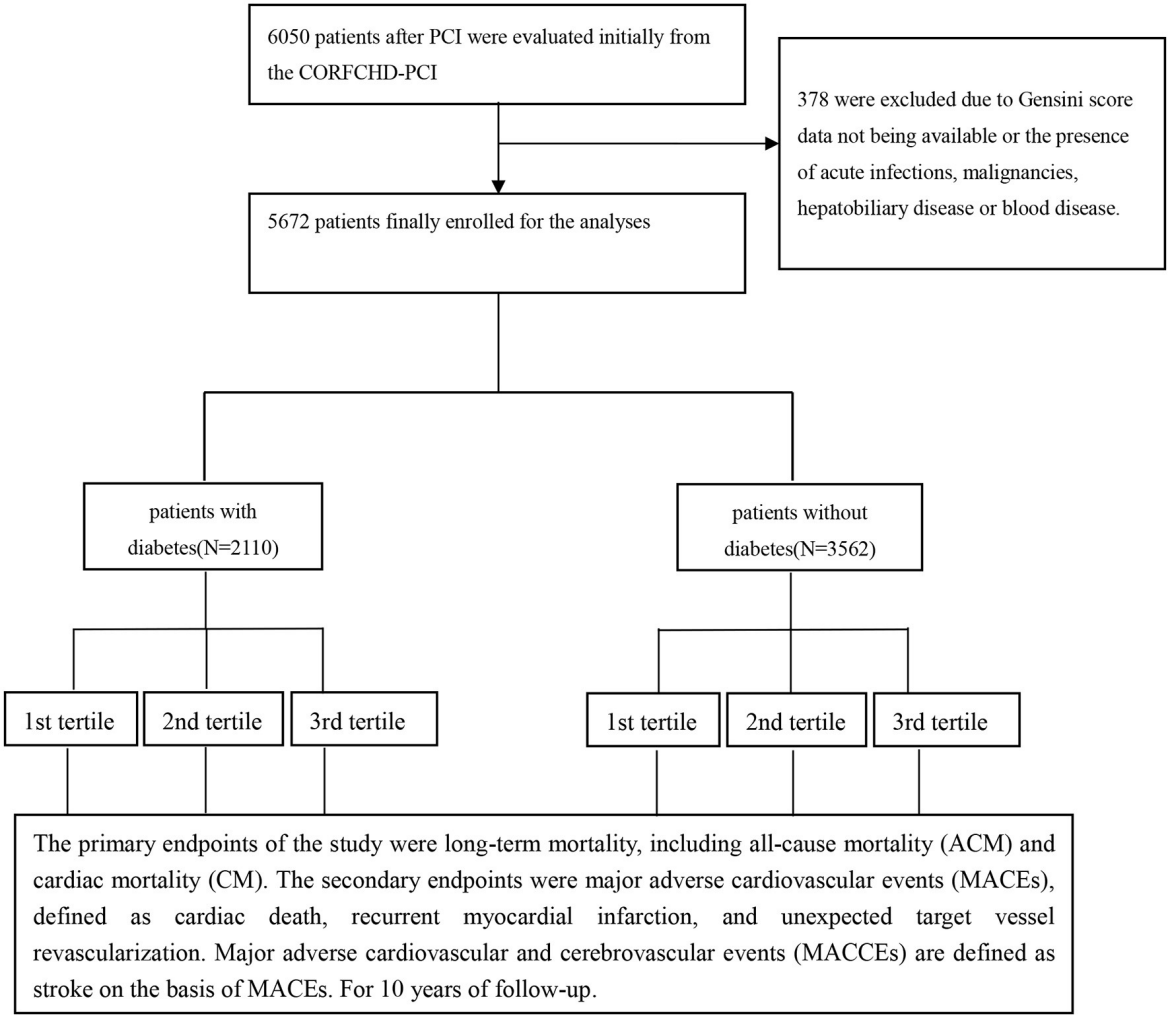
The flowchart of participants' inclusion.

All patients were from the Clinical Outcomes and Risk Factors of Patients with Coronary Heart Disease after PCI (CORFCHD-PCI) study, which is a large single-center retrospective cohort study based on case data and follow-up records in the First Affiliated Hospital of Xinjiang Medical University. Design details have been registered at http://www.Chictr.org.cn (ID: ChiCTR-ORC-16010153). In brief, the CORFCHD-PCI study aims to evaluate and analyze the clinical outcomes and risk factors of patients with CAD after PCI. This cohort study included patients with CAD who underwent PCI at the First Affiliated Hospital of Xinjiang Medical University from January 2008 to December 2017. Demographic data, clinical characteristics, medical history, home medications, risk factors, blood samples, biochemical data, electrocardiogram data, echocardiography data, coronary angiography, and PCI procedures, and short-term and long-term outcomes were also collected. A total of 6,050 patients with CAD who underwent PCI in the CORFCHD-PCI study were evaluated initially. Three hundred and seventy-eight were excluded due to Gensini score data not being available or the presence of acute infections, malignancies, hepatobiliary disease, or blood disease. Finally, 5,672 patients were enrolled in this study, such as 2,110 patients with diabetes and 3,562 patients without diabetes. Diabetes was defined as either a previous diagnosis of diabetes treated with pharmacologic or non-pharmacologic measure, or new diabetes was defined according to the American Diabetes Association as the history of either presence of classic symptoms of diabetes with an unequivocal elevation of plasma glucose (2-h post-prandial or random of ≥200 mg/dl), fasting plasma glucose elevation on ≥126 mg/dl during hospitalization, or hemoglobin A1C ≥6.5% (48 mmol/mol) (10). Hypertension was defined as repeated (at least two times in different circumstances) blood pressure measurements ≥140/90 mmHg and was assumed to be present in patients taking anti-hypertensive drugs. Tobacco smoking was categorized based on the current smoking status (non-smoker or past smoker/current smoker), duration of smoking (non-smoker <20 years and ≥20 years), and the current number of cigarettes smoked per day (0 cigarettes per day, <20 cigarettes per day, and ≥20 cigarettes per day). Current and past smokers were defined as smokers and were compared to non-smokers. Alcohol drinking was evaluated by frequency (<1 time per week and ≥1 time per week) and by the amount of alcohol consumed at a time (<1 beer bottle and ≥1 beer bottle). Generally, a bottle of beer contains 4.5% of alcohol per 100 ml. Alcohol drinking was defined as alcohol consumption ≥1 time per week compared to alcohol consumption <1 time per week. Figure 1 shows the flowchart of the inclusion and exclusion criteria used in the selection of participants. The study protocol was approved by the Ethics Committee of the First Affiliated Hospital of Xinjiang Medical University. Because of the retrospective design of the study, the need to obtain informed consent from eligible patients was waived by the ethics committee.

### Assessment of Coronary Angiography

The coronary angiography was performed for all enrolled individuals, and the results were analyzed by at least two interventional physicians. The severity of CAD was evaluated by the Gensini score assessment system and scored by two independent senior cardiologists. The degree of stenosis and the coronary artery lesion site were scored as follows: 1 point for ≤ 25% narrowing, 2 points for 26–50% narrowing, 4 points for 51–75% narrowing, 8 points for 76–90% narrowing, 16 points for 91–99% narrowing, and 32 points for total occlusion. Thereafter, each lesion score is multiplied by a factor that takes into account the importance of the lesion's position in the coronary circulation (5 for the left main coronary artery, 2.5 for the proximal segment of the left anterior descending coronary artery, 2.5 for the proximal segment of the circumflex artery, 1.5 for the mid-segment of the left anterior descending coronary artery, 1.0 for the right coronary artery, the distal segment of the left anterior descending coronary artery, the posterolateral artery, and the obtuse marginal artery, and 0.5 for other segments). Finally, the Gensini score was calculated by summation of the individual coronary segment scores (4, 11). The patients were classified into three groups according to the tertile of Gensini score: first tertile (Gensini score <11 points), second tertile (Gensini score 11–38 points), third tertile (Gensini score >38 points).

### Endpoints

The primary endpoints of the study were long-term mortality, such as all-cause mortality (ACM) and cardiac mortality (CM). The secondary endpoints were major adverse cardiovascular events (MACEs), defined as cardiac death, recurrent myocardial infarction (MI), and unexpected target vessel revascularization. Target vessel revascularization was defined as any repetitive revascularization in a treated vessel where there was stenosis of at least a 50% diameter in the presence of ischemic signs or symptoms or stenosis of at least 70% in the absence of ischemic signs or symptoms. Major adverse cardiovascular and cerebrovascular events are defined as a stroke on the basis of MACEs.

### Follow-Up

In our center, all of the patients who underwent PCI will receive regular follow-up after discharge at the end of 1 month, 3 months, 6 months, 1 year, 3 years, and 5 years. The follow-up was conducted by telephone contact, follow-up letter, or outpatient interviews. During the follow-up duration, an independent group of clinical physicians carefully checked and verified all events. To obtain high-quality data, before the study, we performed investigator training. All the questionnaire fillings were performed blindly, and the telephone recordings were performed in accordance with uniform criteria. The compliance of the drugs and adverse events were assessed at every visit for clinical follow-up. All the patients who underwent PCI were followed up for 31.0 (interquartile range, IQR: 30.0) months.

### Statistical Analyses

The continuous data of normal distribution are presented as the mean ± SD, the differences between groups were compared by analysis of variance. If the difference between groups was statistically significant, further pairwise comparison was performed by the Fisher Least Significant Difference (LSD) method. The continuous data of non-normal distribution are presented as median (IQR), the differences between groups were compared by Kruskal-Wallis test or H-test. If the difference between groups was statistically significant, the Dunn method was further used for multiple comparisons. Categorical data are presented as the frequencies and percentages, the differences between groups were compared by the chi-square test. Based on the tertiles of Gensini score, the enrolled patients were classified into three groups: first tertile (Gensini score <11 points), second tertile (Gensini score 11–38 points), and third tertile (Gensini score >38 points). Kaplan-Meier analysis was used for cumulative incidence rates of long-term outcomes, and the log-rank test was used for comparisons between groups. Multivariable analysis was performed to assess the prognostic value of the Gensini score for adverse outcomes after adjusting for confounders. Hazard ratios (HRs) and 95% CIs were calculated. Interaction and stratified analyses were conducted according to DM status (with or without). All of the analyses were performed using SPSS22.0 for Windows statistical software (SPSS Inc., Chicago, IL, USA) and the statistical software packages R (The R Foundation; http://www.r-project.org; version 3.6.2). *p* < 0.05 was considered statistically significant for all comparisons.

## Results

### Baseline Data

A total of 5,672 patients with CAD who underwent PCI, such as 2,110 patients with diabetes and 3,562 patients without diabetes, were divided into three groups according to Gensini score tertiles: first tertile (Gensini score <11 points; *n* = 1,846), second tertile (Gensini score 11–38 points; *n* = 1,956), third tertile (Gensini score >38 points; *n* = 1,870). As shown in Table 1, there are significant differences between the three groups for several variables, such as gender, age, smoking, family history of CAD, hypertension, and therapy with ARB or ACEI, SCr, and left ventricular ejection fraction (LVEF; all *p* < 0.05). In addition, several characteristics of lesions and some PCI parameters between these three groups show significant differences, such as LM, CTO, MVD, and number of diseased vessels, as shown in Table 2.

**Table 1:** Baseline characteristics of patients.

**Variables**	**Gensini score**			**χ^2^/*F***	***P*-value**
	**<11 points**	**11–38 points**	**>38 points**		
**CAD (*****N*** **= 5,672)**	***n*** **= 1,815**	***n*** **= 1,916**	***n*** **= 1,941**		
CCB, *n* (%)	234 (12.9)	214 (11.2)	207 (10.8)	4.762	0.092
β-Blockers, *n* (%)	706 (39.0)	793 (41.5)	801 (41.6)	3.224	0.199
ARB or ACEI, *n* (%)	374 (20.7)	430 (22.5)	491 (25.5)	12.390	**0.002**
Statins, *n* (%)	996 (55.3)	1,020 (53.7)	1,068 (55.6)	1.592	0.451
Smoking, *n* (%)	697 (38.4)	786 (41.0)	809 (41.7)	4.637	0.098
Drinking, *n* (%)	540 (29.8)	595 (31.1)	531 (28.6)	3.562	0.086
Family history of CAD, *n* (%)	192 (10.6)	244 (12.7)	279 (14.4)	12.309	**0.002**
Hypertension, *n* (%)	721 (39.7)	836 (43.6)	863 (44.5)	9.710	**0.008**
Age (years)	59.50 ± 10.74^a^	59.60 ± 10.86^a^	59.45 ± 10.99^a^	0.088	0.916
Gender, male, *n* (%)	1,286 (22.7)	1,515 (26.7)	1,426 (25.1)	19.664	**<0.001**
BUN (mmol/L)	5.48 ± 1.68^a^	5.52 ± 1.65^a^	5.55 ± 1.72^a^	0.783	0.457
SCr (μmol/L)	74.44 ± 21.98^a^	75.5 ± 18.55^a^	77.74 ± 20.31^b^	12.634	**<0.001**
TG (mmol/L)	1.90 ± 1.21^a^	1.91 ± 1.28^a^	1.90 ± 1.32^a^	0.046	0.955
TC (mmol/L)	3.93 ± 1.09^a^	3.97 ± 1.14^a^	3.97 ± 1.11^a^	0.819	0.441
HDL-C (mmol/L)	1.03 ± 0.52^a^	1.02 ± 0.44^a^	1.01 ± 0.44^a^	0.938	0.391
LDL-C (mmol/L)	2.45 ± 0.91^a^	2.47 ± 0.92^a^	2.45 ± 0.91^a^	0.360	0.698
ApoA1 (mmol/L)	1.17 ± 0.32^a^	1.17 ± 0.32^a^	1.16 ± 0.32^a^	0.383	0.682
ApoB (mmol/L)	0.84 ± 0.35^a^	0.86 ± 0.42^a^	0.85 ± 0.39^a^	1.510	0.221
Lp(a) (mmol/L)	217.99 ± 171.25^a^	223.04 ± 176.66^a^	220.11 ± 175.79^a^	0.369	0.692
LVEDD (mm)	49.94 ± 5.50^a^	50.16 ± 5.55^a^	49.89 ± 5.50^a^	1.135	0.321
LVEF (%)	61.30 ± 6.84^a^	60.9 ± 7.08^a^	60.94 ± 7.22^a^	1.919	0.147
**CAD without diabetes (*****N*** **= 3,562)**	***n*** **= 1,251**	***n*** **= 1,208**	***n*** **= 1,103**		
CCB, *n* (%)	166 (13.3)	132 (11.0)	120 (11.6)	4.185	0.123
β-Blockers, *n* (%)	482 (38.7)	502 (41.7)	458 (41.8)	3.234	0.199
ARB or ACEI, *n* (%)	255 (20.5)	260 (21.6)	279 (25.5)	9.106	**0.011**
Statins, *n* (%)	685 (55.2)	633 (52.8)	594 (54.3)	1.452	0.484
Smoking, *n* (%)	477 (38.1)	506 (41.9)	485 (44.0)	8.599	**0.014**
Drinking, *n* (%)	374 (29.9)	385 (31.9)	307 (27.8)	4.483	0.106
Family history of CAD, *n* (%)	121 (9.7)	150 (12.4)	160 (14.5)	13.050	**0.001**
Hypertension, *n* (%)	439 (35.1)	484 (40.1)	441 (40.0)	8.364	**0.015**
Age (years)	59.16 ± 10.83^a^	59.17 ± 11.06^a^	58.83 ± 11.16^a^	0.350	0.704
Gender, male, *n* (%)	1,009 (23.5)	1,170 (27.2)	1,066 (24.8)	28.309	**<0.001**
BUN (mmol/L)	5.45 ± 1.61^a^	5.52 ± 1.61^a^	5.43 ± 1.60^a^	1.125	0.325
SCr (μmol/L)	73.76 ± 20.74^a^	75.66 ± 18.14^b^	77.52 ± 17.92^c^	11.092	**<0.001**
TG (mmol/L)	1.80 ± 1.06^a^	1.84 ± 1.21^a^	1.76 ± 1.03^a^	1.404	0.246
TC (mmol/L)	3.87 ± 1.06^a^	3.93 ± 1.12^a^	3.91 ± 1.05^a^	0.875	0.417
HDL-C (mmol/L)	1.03 ± 0.56^a^	1.0150 ± 0.44^a^	1.01 ± 0.46^a^	0.684	0.505
LDL-C (mmol/L)	2.43 ± 0.92^a^	2.45 ± 0.92^a^	2.44 ± 0.89^a^	0.158	0.854
ApoA1 (mmol/L)	1.15 ± 0.30^a^	1.17 ± 0.34^a^	1.16 ± 0.34^a^	1.031	0.357
ApoB (mmol/L)	0.82 ± 0.34^a^	0.85 ± 0.43^a^	0.84 ± 0.39^a^	1.631	0.196
LP(a) (mmol/L)	219.92 ± 167.47^a^	226.87 ± 177.35^a^	218.28 ± 171.35^a^	0.767	0.465
LVEDD (mm)	50.07 ± 5.68^a^	50.12 ± 5.63^a^	49.88 ± 5.57^a^	0.528	0.590
LVEF (%)	61.18 ± 7.01^a^	60.99 ± 7.18^a^	61.01 ± 7.32^a^	0.235	0.790
**CAD with diabetes (*****N*** **= 2,110)**	***n*** **= 564**	***n*** **= 708**	***n*** **= 838**		
CCB, *n* (%)	68 (12.1)	82 (11.6)	87 (10.5)	1.038	0.595
β-Blockers, *n* (%)	224 (39.9)	291 (41.2)	343 (41.2)	0.317	0.853
ARB or ACEI, *n* (%)	119 (21.2)	170 (24.2)	212 (25.5)	3.470	0.176
Statins, *n* (%)	311 (55.6)	387 (55.3)	474 (57.2)	0.677	0.713
Smoking, *n* (%)	220 (39.0)	280 (39.5)	324 (38.7)	0.127	0.939
Drinking, *n* (%)	166 (29.4)	210 (29.7)	218 (26.0)	3.148	0.207
Family history of CAD, *n* (%)	71 (12.6)	94 (13.3)	119 (14.2)	0.782	0.676
Hypertension, *n* (%)	282 (50.0)	352 (49.7)	422 (50.4)	0.064	0.969
Age (years)	60.28 ± 10.51^a^	60.32 ± 10.49^a^	60.26 ± 10.70^a^	0.006	0.994
Gender, male, *n* (%)	277 (20.2)	345 (25.1)	360 (26.2)	0.908	0.635
BUN (mmol/L)	5.54 ± 1.82^a^	5.51 ± 1.71^a^	5.71 ± 1.86^a^	2.701	0.067
SCr (μmol/L)	75.91 ± 24.40^ab^	75.27 ± 19.23^b^	78.01 ± 23.04^c^	3.181	**0.042**
TG (mmol/L)	2.10 ± 1.46^a^	2.03 ± 1.39^a^	2.09 ± 1.59^a^	0.384	0.681
TC (mmol/L)	4.05 ± 1.13^a^	4.04 ± 1.16^a^	4.04 ± 1.17^a^	0.004	0.996
HDL-C (mmol/L)	1.02 ± 0.43^a^	1.02 ± 0.42^a^	1.01 ± 0.43^a^	0.287	0.750
LDL-C (mmol/L)	2.49 ± 0.88^a^	2.51 ± 0.91^a^	2.47 ± 0.93^a^	0.360	0.698
ApoA1 (mmol/L)	1.19 ± 0.35^a^	1.16 ± 0.28^a^	1.16 ± 0.28^a^	2.827	0.059
ApoB (mmol/L)	0.89 ± 0.38^a^	0.88 ± 0.42^a^	0.87 ± 0.39^a^	0.473	0.623
LP(a) (mmol/L)	213.97 ± 179.00^a^	216.74 ± 175.47^a^	222.45 ± 181.39^a^	0.405	0.667
LVEDD (mm)	49.63 ± 5.08^a^	50.22 ± 5.41^a^	49.91 ± 5.41^a^	1.670	0.189
LVEF (%)	61.69 ± 6.45^a^	60.76 ± 6.90^b^	60.85 ± 7.09^bc^	3.055	**0.047**

a,b,c *After comparing the means between groups, the test was performed. If there are the same letters between the groups, the difference is not statistically significant (P > 0.05); if the letters are different between the groups, the difference is statistically significant (P <0.05). TG, triglyceride; TC, total cholesterol; LDL-C, low-density lipoprotein cholesterol; HDL-C, high-density lipoprotein cholesterol; ApoA1, apolipoprotein A1; ApoB, apolipoprotein B; Lp(a), lipoprotein a; Scr, serum creatinine; BUN, blood urea nitrogen; ACEI, angiotensin-converting enzyme inhibitor ARB, angiotensin receptor blocker; CCB, calcium channel blocker; LVEDD, left ventricular end diastolic diameter; LVEF, left ventricular ejection function. In order to make the number of patients in different groups more clearly visible*.

**Table 2:** Procedural characteristics of patients in the total population.

**Variables**	**Gensini score**			***X*^2^/*F***	***P*-value**
	**<11 points**	**11–38 points**	**>38 points**		
	***n* = 1,815**	***n* = 1,916**	***n* = 1,941**		
LM, *n* (%)	78 (4.3)	128 (6.7)	205 (10.6)	56.075	**<0.0001**
CTO, *n* (%)	249 (13.7)	321 (16.8)	788 (40.6)	453.960	**<0.0001**
MVD, *n* (%)	889 (49.0)	1,346 (70.3)	1,522 (78.4)	383.333	**<0.0001**
DES, *n* (%)	1,723 (95.0)	1,800 (93.9)	1,831 (94.3)	1.935	0.380
Pre-expansion, *n* (%)	1,593 (87.8)	1,645 (85.9)	1,675 (86.3)	3.386	0.184
Post-expansion, *n* (%)	1,140 (62.8)	1,194 (62.3)	1,216 (62.6)	0.114	0.945
Number of stents	1.043 ± 0.210^a^	1.043 ± 0.227^a^	1.038 ± 0.216^a^	0.376	0.689
Number of diseased vessels	1.716 ± 0.810^a^	2.065 ± 0.809^b^	2.305 ± 0.802^c^	251.493	**<0.0001**
Diameter of stents, (mm)	2.857 ± 0.369^a^	2.832 ± 0.366^b^	2.861 ± 0.386^ac^	3.529	0.029
Length of stents, (mm)	28.193 ± 6.958^a^	27.745 ± 7.011^a^	28.084 ± 6.860^a^	2.131	0.119
Expansion pressure, (atm)	11.855 ± 3.407^a^	11.793 ± 2.486^a^	11.899 ± 2.565^a^	0.322	0.725

a,b,c *After comparing the means between groups, the test was performed. If there are the same letters between the groups, the difference is not statistically significant (P > 0.05); if the letters are different between the groups, the difference is statistically significant (P <0.05). LM, left main coronary artery disease; CTO, chronic total occlusion coronary artery disease; MVD, multivessel disease; DES, drug-eluting stent; pre-expansion, before the stent is implanted, the pressure of the balloon is used to expand the coronary artery stenosis; post-expansion, after the stent is implanted, the pressure of the balloon is used to fully expand the stent. In order to make the number of patients in different groups more clearly visible*.

### Clinical Outcomes

In the total population, during the median follow-up period of 31.0 (IQR: 30.0) months, there were 300 cases of ACM. In total, the incidence of ACM in the first tertile was 68 (3.7%), the second tertile was 90 (4.7%), and the third tertile was 142 (7.3%). There was a significant difference in the ACM incidence among these three groups (*p* < 0.001). We also found that CM occurred in 243 patients: 51 (2.8%) in the first tertile, 74 (3.9%) in the second tertile, 118 (6.1%) in the third tertile. There was a significant difference in the CM incidence among these three groups (*p* < 0.001). Regarding the incidence of MACEs and MACCEs, there are significant differences among the three groups (all *p* < 0.001). In the 3,562 CAD patients without diabetes and 2,110 CAD patients with diabetes, we found that there are significant differences among these three groups in the incidence of ACM, CM, MACEs, and MACCEs (all *p* < 0.05), as shown in Table 3. However, There were no significant differences among the groups in the incidence of stroke, readmission, recurrent MI, target vessel revascularization, and bleeding events (all *p* > 0.05), only in people with diabetes, the incidence of heart failure (HF) is statistically significant (*p* = 0.035; Supplementary Table 1).

**Table 3:** Clinical outcomes and Gensini score according to tertiles.

**Clinical outcomes**	**Gensini score**			***X* ^2^**	***P*-value**
	**<11 points**	**11–38 points**	**>38 points**		
**CAD (*****N*** **= 5,672)**	***n*** **= 1,815**	***n*** **= 1,916**	***n*** **= 1,941**		
ACM	68 (3.7)	90 (4.7)	142 (7.3)	25.877	<0.001
CM	51 (2.8)	74 (3.9)	118 (6.1)	25.706	<0.001
MACEs	185 (10.2)	246 (12.8)	315 (16.2)	30.164	<0.001
MACCEs	207 (11.4)	275 (14.4)	336 (17.3)	26.517	<0.001
**CAD without diabetes (*****N*** **= 3,562)**	***n*** **= 1,251**	***n*** **= 1,208**	***n*** **= 1,103**		
ACM	53 (4.2)	59 (4.9)	71 (6.4)	6.064	0.048
CM	40 (3.2)	49 (4.1)	60 (5.4)	7.426	0.024
MACEs	131 (10.5)	150 (12.4)	164 (14.9)	10.376	0.006
MACCEs	146 (11.7)	165 (13.7)	177(16.0)	9.499	0.009
**CAD with diabetes (*****N*** **= 2,110)**	***n*** **= 564**	***n*** **= 708**	***n*** **= 838**		
ACM	15 (2.7)	31 (4.4)	71 (8.5)	24.517	<0.001
CM	11 (2.0)	25 (3.5)	58 (6.9)	21.707	<0.001
MACEs	54 (9.6)	96 (13.6)	151 (18.0)	20.090	<0.001
MACCEs	61 (10.8)	110 (15.5)	159 (19.0)	17.014	<0.001

**Figure 2:**
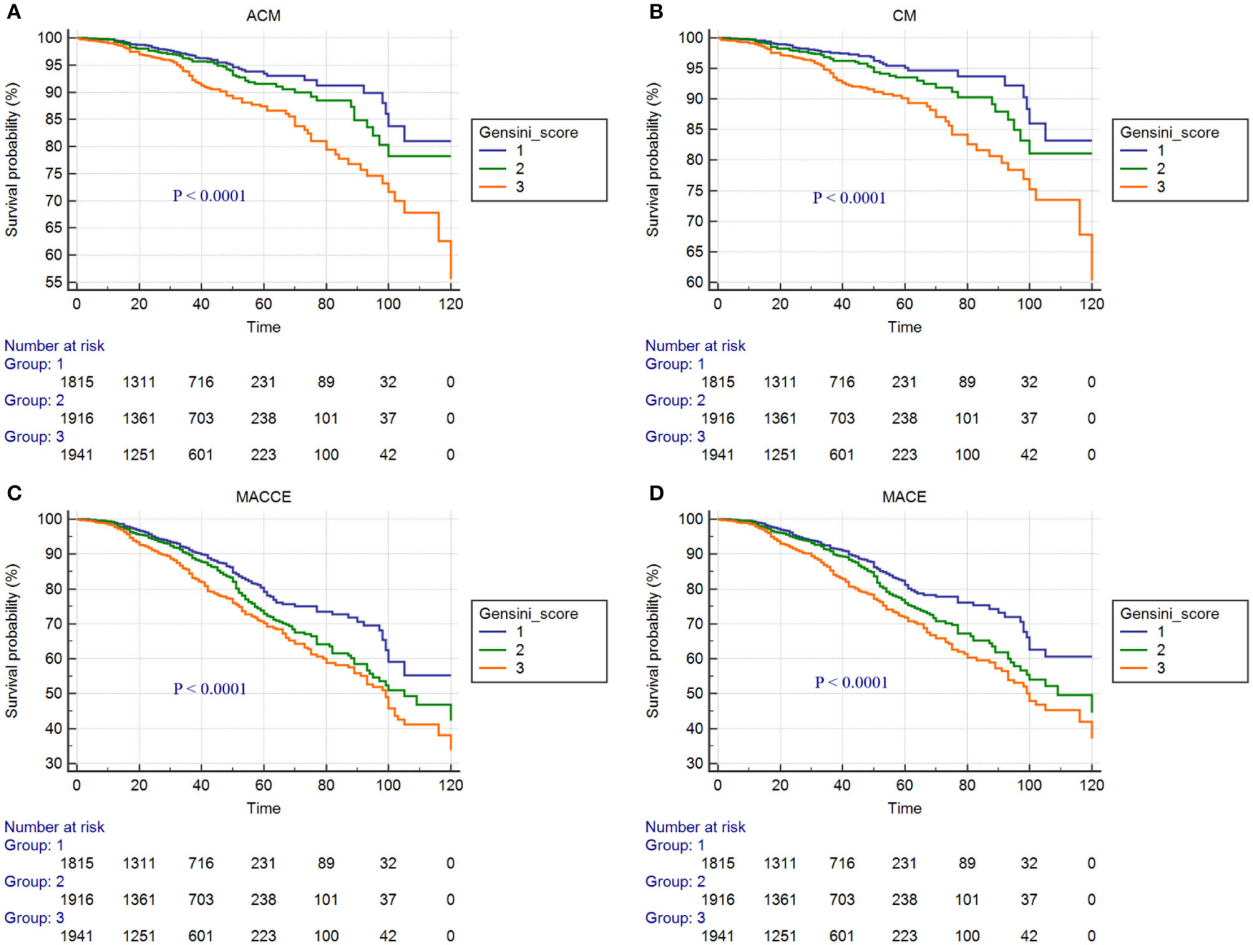
Cumulative Kaplan-Meier estimates of the time to the first adjudicated occurrence of primary endpoints and secondary endpoints: **(A)** ACM; **(B)** CM; **(C)** MACCEs; **(D)** MACEs. (In the total population).

**Figure 3:**
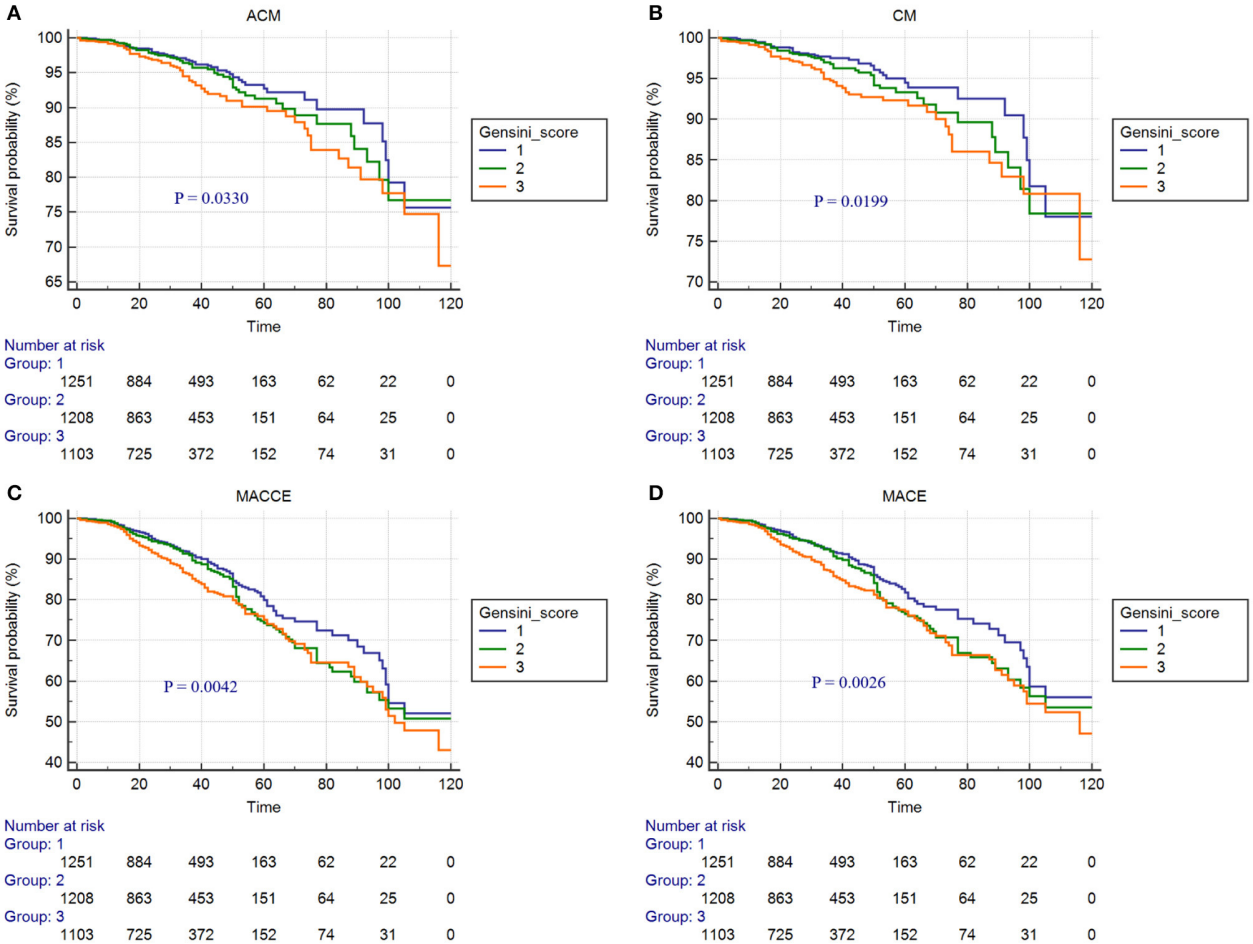
Cumulative Kaplan-Meier estimates of the time to the first adjudicated occurrence of primary endpoints and secondary endpoints: **(A)** ACM; **(B)** CM; **(C)** MACCEs; **(D)** MACEs. (In the 3,562 CAD patients without diabetes).

**Figure 4:**
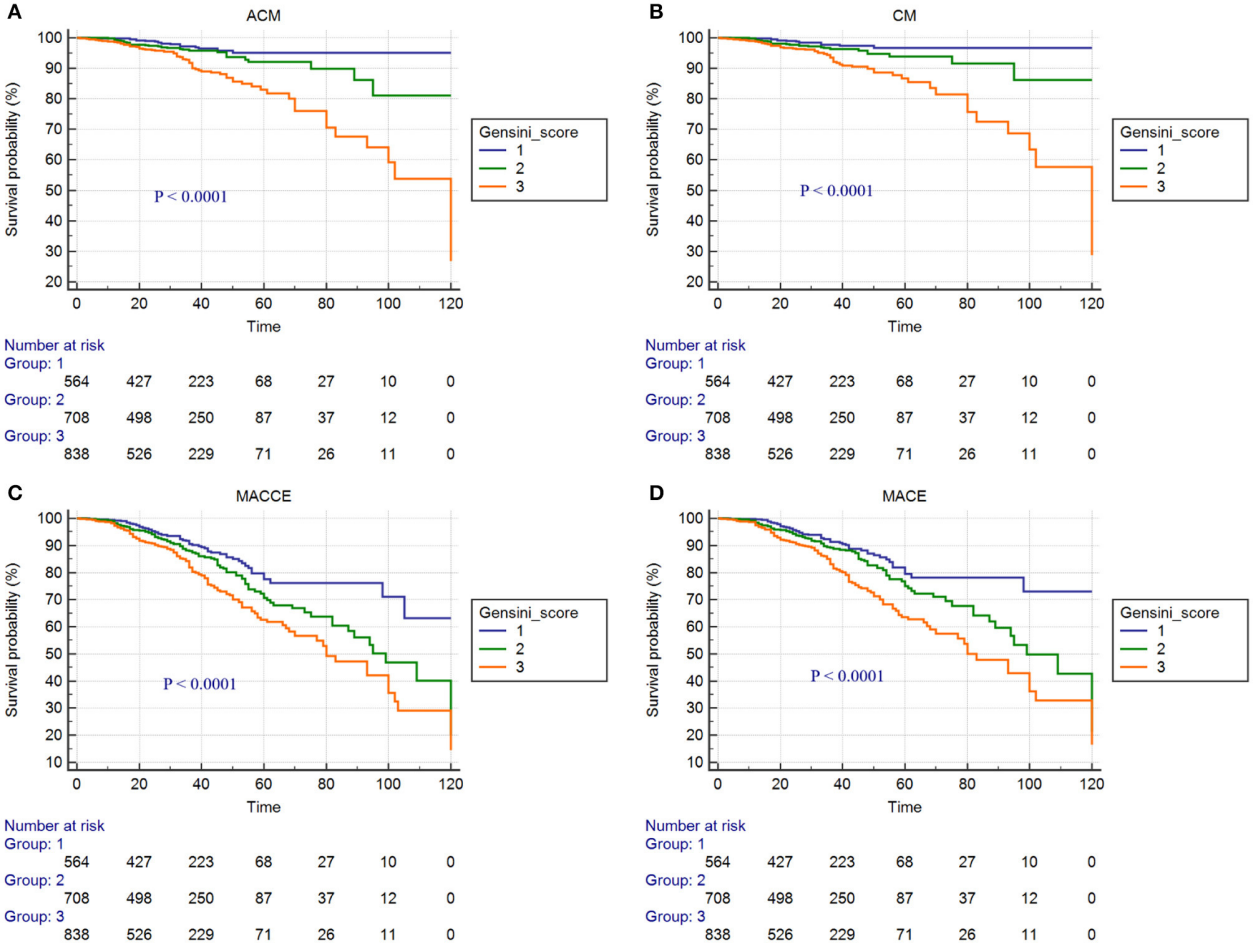
Cumulative Kaplan-Meier estimates of the time to the first adjudicated occurrence of primary endpoints and secondary endpoints: **(A)** ACM; **(B)** CM; **(C)** MACCEs; **(D)** MACEs. (In the 2,110 CAD patients with diabetes).

Kaplan-Meier curves for Gensini score divided by tertiles and adverse outcomes are shown in Figures 2–4. In the total population, patients in the third tertile with Gensini score >38 points and the second tertile with Gensini scores 11–38 points showed significantly higher event rates for ACM (7.3 vs. 3.7% and 4.7 vs. 3.7%), CM (6.1 vs. 2.8% and 3.9 vs. 2.8%), MACEs (16.2 vs. 10.2% and 12.8 vs. 10.2%), and MACCEs (17.3 vs. 11.4% and 14.4 vs. 11.4%) compared with patients in the first tertile with Gensini score <11 points. These differences were also found in both patients without diabetes and patients with diabetes (data not shown).

### Multivariate Cox Regression Analysis in Different Clinical Outcomes

Multivariable analysis was performed to assess the prognostic value of the Gensini score for adverse outcomes after adjusting for age, gender, smoking, family history of CAD, hypertension, SCr, LVEF, and therapy with ARB or ACEI. After multivariate Cox regression analyses, in the total population the respective risks of ACM, CM, MACEs, and MACCEs was increased 141.5% (HR = 2.415, 95% CI: 1.767–3.301, *p* < 0.001), 164.9% (HR = 2.649, 95% CI: 1.850–3.793, *p* < 0.001), 79.2% (HR = 1.792, 95% CI: 1.471–2.818, *p* < 0.001), and 76.3% (HR = 1.763, 95% CI: 1.460–2.129, *p* < 0.001) in the third tertile compare to those in the first tertile, as shown in Table 4. Cox regression stratified analysis shows that the correlation between Gensini score and the risk of ACM, CM, MACE, MACCE was statistically significant in the diabetic status (with or without) (all interactions *p* < 0.05). In the population with diabetes, compared with the first tertile, the risks of ACM were increased 248.5% (HR = 3.485, 95% CI: 1.973–6.154, *p* < 0.001) in the third tertile and 69.0% (HR = 1.690, 95% CI: 0.904–3.161, *p* < 0.001) in second tertile. In the population without diabetes, compared with the first tertile, the risks of ACM were increased 89.9% (HR = 1.899, 95% CI: 1.285–2.807, *p* = 0.001) in the third tertile and 28.4% (HR = 1.284, 95% CI: 0.855–1.928, *p* = 0.229) in second tertile. The results of the interaction test showed that the relationship between Gensini score and ACM was different in diabetes status (with or without) (*p* = 0.011). Compared with non-diabetic people, the higher the Gensini score in diabetic people, the higher the risk of ACM. These differences are also found in other clinical outcomes (CM, MACEs, and MACCEs) (data not shown), as shown in Table 5. In addition, compared with the non-diabetic population, the incidence of ACS was higher in the diabetic population, and the difference was statistically significant (*p* = 0.002; Supplementary Table 2).

**Table 4:** Multivariable Cox regression analysis of ACM, CM, MACEs, and MACCEs.

**Variables**	**Total population (ACM)**			**Total population (CM)**			**Total population (MACEs)**			**Total population (MACCEs)**		
	***Z*-values**	***P*-values**	**HR (95%CI)**	***Z*-values**	***P*-values**	**HR (95%CI)**	***Z*-values**	***P*-values**	**HR (95%CI)**	***Z*-values**	***P*-values**	**HR (95%CI)**
Gender, male	0.018	0.894	1.022 (0.747–1.397)	0.138	0.710	1.069 (0.753–1.516)	1.014	0.314	0.898 (0.728–1.107)	1.790	0.181	0.873 (0.715–1.065)
Age	12.331	<0.001	1.022 (1.010–1.035)	3.555	0.059	1.013 (0.999–1.027)	0.142	0.706	0.999 (0.991–1.006)	0.113	0.737	1.001 (0.994–1.009)
Smoking	0.996	0.318	0.868 (0.657–1.146)	2.550	0.110	0.774 (0.565–1.060)	6.347	0.012	0.797 (0.667–0.951)	9.765	0.002	0.763 (0.644–0.904)
Family history of CAD	3.601	0.058	0.591 (0.343–1.017)	1.814	0.178	0.677 (0.384–1.194)	2.053	0.152	1.201 (0.935–1.541)	2.216	0.137	1.200 (0.944–1.526)
Hypertension	5.349	0.021	1.341 (1.046–1.720)	3.045	0.081	1.285 (0.970–1.702)	12.867	<0.001	1.341 (1.142–1.574)	14.607	<0.001	1.348 (1.157–1.571)
ARB or ACEI	36.213	<0.001	0.099 (0.047–0.211)	28.900	<0.001	0.126 (0.059–0.268)	0.908	0.341	0.910 (0.748–1.105)	0.753	0.386	0.921 (0.765–1.109)
SCr	5.569	0.018	1.006 (1.001–1.011)	9.670	0.002	1.008 (1.003–1.013)	0.631	0.427	1.002 (0.998–1.005)	1.321	0.250	1.002 (0.999–1.006)
LVEF	0.585	0.444	1.007 (0.990–1.024)	0.001	0.981	1.000 (0.982–1.019)	0.191	0.662	1.002(0.991–1.014)	1.168	0.280	1.006 (0.995–1.017)
**Gensin score** (** <11 points as reference)**												
11–38 points	3.809	0.051	1.400 (0.999–1.963)	4.383	0.036	1.515 (1.027–2.234)	3.916	0.048	1.232 (1.002–1.516)	5.740	0.017	1.272 (1.045–1.549)
>38 points	30.611	<0.001	2.415 (1.767–3.301)	28.284	<0.001	2.649 (1.850–3.793)	33.822	<0.001	1.792 (1.472–2.181)	34.717	<0.001	1.763 (1.460–2.129)

*SCr, serum creatinine; ACEI, angiotensin-converting enzyme inhibitor ARB, angiotensin receptor blocker; LVEF, left ventricular ejection function*.

**Table 5:** Cox regression stratified analysis of Gensini score and the risk of ACM, CM, MACEs, MACCEs.

**Clinical outcomes/factors**		**Gensini score HR (95%CI)**			**Interaction *P*-value**
		**<11 points**	**11–38 points**	**>38 points**	
**ACM**					
Diabetes	With	1	1.690 (0.904–3.161)	3.485 (1.973–6.154)	0.011
	Without	1	1.284 (0.855–1.928)	1.899 (1.285–2.807)	
**CM**					
Diabetes	With	1	1.817 (0.882–3.744)	3.604 (1.866–6.963)	0.007
	Without	1	1.401 (0.879–2.236)	2.151 (1.378–3.365)	
**MACEs**					
Diabetes	With	1	1.535 (1.076–2.189)	2.302 (1.649–3.215)	0.008
	Without	1	1.068 (0.824–1.384)	1.481 (1.152–1.904)	
**MACCEs**					
Diabetes	With	1	1.598 (1.145–2.231)	2.198 (1.600–3.018)	0.010
	Without	1	1.095 (0.854–1.403)	1.498 (1.176–1.907)	

*Multivariable analysis was performed to assess the prognostic value of Gensini score for adverse outcomes after adjusting for age, gender, smoking, family history of CAD, hypertension, Scr, and LVEF, as well as therapy with ARB or ACEI*.

## Discussion

In the present study, we demonstrate that Gensini score in patients with CAD treated with PCI was an independent predictor of adverse outcomes over up to 10 years of follow-up. The present results indicate the strong relationship between Gensini score and adverse outcomes over a follow-up period of up to 10 years in patients with CAD who underwent PCI. Recent research performed by Yokokawa et al. (11) reported that a high Gensini score after PCI was associated with higher CM in HF patients, suggesting that residual coronary atherosclerotic burden might lead to a higher risk of cardiac events. In our study, we enrolled 5,672 patients with CAD who underwent PCI and analyzed four different clinical outcomes: ACM, CM, MACEs, MACCEs, and further verified that the Gensini score was an independent predictor of adverse outcomes of patients with CAD after PCI. The present results were compatible with those of some previous studies. A study by Reynolds et al. (12) investigated that CAD severity was a highly significant predictor of ACM, MI, CV death, and other five adverse clinical outcomes, independent of ischemia severity and other clinical predictors.

The main purpose of coronary angiography is to determine whether there is CAD, assess the degree of coronary artery stenosis, and treat diseased vessels. The morphology and degree of stenosis of coronary artery lesions determine the choice of the treatment plan. At present, there are many scoring systems for quantitative analysis of coronary artery lesions, among which the Gensini score and SYNTAX score are more commonly used. They have different emphases, and each has its own advantages and disadvantages (13). Gensini score fully considers the number, location, and degree of coronary artery lesions and is a relatively scientific evaluation standard. The scoring system divides the coronary artery into 14 segments, each of which has its own weighting coefficient. In particular, the left main artery, the proximal and middle segments of the left anterior descending branch dominate the blood supply to the left ventricle, so they have a higher weighting coefficient (14). At the same time, the Gensini score has been widely integrated into various clinical studies. Currently, the most reported research is Gensini score combined with certain biochemical indicators to assess the severity of CAD and predict long-term outcomes. A study by Duran et al. suggested that serum uric acid levels were positively correlated with the Gensini scores in patients with the acute coronary syndrome (ACS). The higher the serum uric acid level, the greater the number of coronary artery lesions, the more severe the stenosis, and even total occlusion. Multivariate analysis showed that serum uric acid level was an independent risk factor for multivessel disease (7). Another study by Chen et al. investigated that the neutrophil-to-lymphocyte ratio (NLR) was an independent predictor of high Gensini score, and NLR was positively correlated with Gensini score. In the ROC curves analysis, the NLR was found to have the largest area under the curve (AUC = 0.63, 95% CI: 0.59–0.67, *p* = 0.000), with an optimal cut-off value of 2.04 (sensitivity: 62.1%, specificity: 54.8%) for predicting a high Gensini score (8). Research performed by Liu et al. reported that the incidence of MACEs in patients with STEMI within 6 months after emergency PCI was 19.36%. Compared with the non-MACEs group, the mean platelet volume (MPV) and Gensini score of the MACEs group were significantly higher. Multivariate Cox analysis showed that MPV and Gensini score were independent risk factors for MACEs in patients with STEMI after emergency PCI (9).

The SYNTAX score is also a commonly used method for quantitative analysis of coronary artery lesions. The scoring method uses the 16-segment method, combining the dominant distribution, lesion location, degree of stenosis, and lesion characteristics to score coronary artery lesions with a diameter of ≥1.5 mm and a degree of stenosis ≥50%. The scoring system refines the following four aspects: dominant distribution, number of lesions, number of diseased vascular segments, and lesion characteristics, mainly including chronic total occlusion lesions, bifurcation lesions, opening lesions, severe tortuosity lesions, >20 mm lesions, calcification lesions, thrombosis lesions, and small vessel lesions (15). The higher the SYNTAX score, the more severe the coronary artery lesions, the worse the prognosis, and the higher the revascularization rate. The SYNTAX score can assist in guiding the choice of reasonable revascularization in patients with three branches lesions or left main artery lesions. The higher the SYNTAX score, the worse the short-term clinical outcome after PCI (16, 17). Wang reported that a study of 2,348 patients with congenital heart disease (CHD) and the SYNTAX score were performed for all enrolled patients before PCI, and then divided into high-risk group, medium-risk group, and low-risk group. The results showed that the differences in ACM and MACEs among the three groups were statistically significant. Multivariate Cox regression analysis showed that SYNTAX score was a risk factor for poor prognosis in patients with CHD after PCI (18).

However, in actual clinical work, the left main artery lesions only account for 3–5%, and the multivessel lesions only account for about 12%. The majority of cases with clear indications for coronary intervention are type A or type B lesions. Therefore, it is complicated and cumbersome to carry out quantitative analysis of coronary artery lesions using SYNTAX score regularly. Because of its simplicity and science, the Gensini score is suitable for the majority of patients with CAD, especially for UA, NSTEMI, and STEMI patients who underwent emergency PCI treatment. It can quickly evaluate coronary artery lesions, identify high-risk patients, and promptly carry out diagnosis and treatment.

In our study, there were significant differences in the incidence of ACM, CM, MACEs, and MACCEs among the three Gensini score groups in patients with CAD who underwent PCI with or without diabetes. Kaplan-Meier curve showed that in the clinical adverse outcomes of ACM, CM, MACEs, and MACEs, the prognosis of patients in the low Gensini score group was better than those in the intermediate Gensini score group and high Gensini score group. After multivariate Cox regression analyses, the risks of ACM, CM, MACEs, and MACCEs increased significantly in the third tertile compared with those in the first tertile, and this result was more pronounced in patients with diabetes. The above results show that, first of all, the higher the Gensini score of patients with CAD after PCI, the greater risk of poor clinical outcomes. Therefore, patients with high Gensini score should be closely followed up and timely adjusted treatment to avoid the occurrence of poor prognosis. Secondly, the risk of adverse prognosis was significantly higher in diabetic patients than in non-diabetic patients. Some previous studies have also proved this point of view. The study of Karayiannides and Norhammar believed that patients with diabetes had higher rates of ACM (9.0 vs. 4.9%; *p* < 0.001) when compared with patients without diabetes. Multivariable regression analysis showed that diabetes was independently associated with increased risk for ACM at 1 year (HR = 1.57; 95% CI: 1.23–2.00; *p* < 0.001) (19). In addition, a multicenter cohort study in South Korea showed that the presence of diabetes and renal failure were strong predictors of MACE and target-vessel revascularization (TVR). After inverse probability of treatment weighting (IPTW) analyses, patients with diabetes had significantly increased rates of 2-year MACE (HR = 2.07, 95% CI: 1.50–2.86; *p* < 0.001) (10). A probable explanation is that patients with diabetes had more CV risk factors than patients without diabetes, some patients with adverse prognoses most likely have a more advanced diabetes disease with longer duration, worse glycemic control, higher risk for hypoglycemia, and underlying macro- and microvascular complications. Finally, our research concluded that age, smoking, hypertension, ARB or ACEI drugs, and Scr were also independent predictors of ACM, CM, MACEs, and MACCEs. It suggests that smoking cessation, blood pressure control, regular use of ARB or ACEI drugs, and protection of renal function have positive effects on improving the long-term prognosis of patients with CAD who underwent PCI. There were several strengths of our study. First, this study is a large single-center retrospective cohort study involving a total of 5,672 patients with CAD who underwent PCI, which improved the statistical power. Second, all patients have undergone long-term follow-up, with the longest experience being 10 years. Compared with previous studies, the follow-up time is the longest. Finally, we analyzed the data with multifaceted methods and provided a comprehensive understanding of the relationship between the Gensini score and clinical outcomes. However, the limitations of our study are also mentioned. The present study is a single-center retrospective cohort design. Therefore, our results need to be further verified by a multicenter, prospective study.

## Conclusion

In conclusion, the present study suggests that the Gensini score is an independent predictor of long-term adverse outcomes in patients with CAD who underwent PCI, and it has a stronger predictive value in the diabetic population. Our results emphasize that patients with high Gensini scores should be closely followed up and timely adjustment of treatment to avoid adverse clinical outcomes.

## Data Availability Statement

The original contributions presented in the study are included in the article/Supplementary Material, further inquiries can be directed to the corresponding author/s.

## Ethics Statement

The studies involving human participants were reviewed and approved by Ethics Committee of the First Affiliated Hospital of Xinjiang Medical University. Written informed consent for participation was not required for this study in accordance with the national legislation and the institutional requirements.

## Author Contributions

K-YW contributed to the literature search, study design, data collection, data analysis and interpretation, and writing of the manuscript. T-TW, Y-YZ, and XX participated in the literature search, study design, data analysis and interpretation, and in the writing of the manuscript. Y-TM conducted the literature search, data analysis and interpretation, and wrote the manuscript. All authors contributed to manuscript revision, read, and approved the submitted version.

## Funding

This work was supported financially by grants from the National Natural Science Foundation of China (81770235).

## Conflict of Interest

The authors declare that the research was conducted in the absence of any commercial or financial relationships that could be construed as a potential conflict of interest.

## Publisher's Note

All claims expressed in this article are solely those of the authors and do not necessarily represent those of their affiliated organizations, or those of the publisher, the editors and the reviewers. Any product that may be evaluated in this article, or claim that may be made by its manufacturer, is not guaranteed or endorsed by the publisher.

## References

[1] Yang X Li J Hu D Chen J Li Y Huang J. Predicting the 10-year risks of atherosclerotic cardiovascular disease in Chinese population: the China-PAR project (prediction for ASCVD risk in China). Circulation. (2016) 134:1430–40. 10.1161/CIRCULATIONAHA.116.022367 27682885

[2] Kwon O Lee JB Ahn JM Kang SJ Lee SW Kim YH. Clinical outcomes of contemporary drug-eluting stents in patients with and without diabetes mellitus: multigroup propensity-score analysis using data from stent-specific, multicenter, prospective registries. Catheter Cardiovasc Interv. (2020) 96:243–52. 10.1002/ccd.28462 31478593

[3] Norhammar A Lagerqvist B Saleh N. Long-term mortality after PCI in patients with diabetes mellitus: results from the Swedish Coronary Angiography and Angioplasty Registry. EuroIntervention. (2010) 5:891–7. 10.4244/EIJV5I8A152 20542773

[4] Gensini GG. A more meaningful scoring system for determining the severity of coronary heart disease. Am J Cardiol. (1983) 51:606. 10.1016/S0002-9149(83)80105-2 6823874

[5] Huang G Zhao JL Du H Lan XB Yin YH. Coronary score adds prognostic information for patients with acute coronary syndrome. Circ J. (2010) 74:490–5. 10.1253/circj.CJ-09-0637 20057158

[6] Sinning C Lillpopp L Appelbaum S Ojeda F Zeller T Schnabel R. Angiographic score assessment improves cardiovascular risk prediction: the clinical value of SYNTAX and Gensini application. Clin Res Cardiol. (2013) 102:495–503. 10.1007/s00392-013-0555-4 23519584

[7] Duran M Kalay N Akpek M Orscelik O Elcik D Ocak A. High levels of serum uric acid predict severity of coronary artery disease in patients with acute coronary syndrome. Angiology. (2012) 63:448–52. 10.1177/0003319711426868 22096206

[8] Chen J Chen MH Li S Guo YL Zhu CG Xu RX. Usefulness of the neutrophil-to-lymphocyte ratio in predicting the severity of coronary artery disease: a Gensini score assessment. J Atheroscler Thromb. (2014) 21:1271–82. 10.5551/jat.25940 25069816

[9] HJ Liu JY. Mean platelet volume combined with Gensini score predicts the short-term outcome of emergency percutaneous coronary intervention in patients with ST-segment elevation myocardial infarction. Chin J Intervent Imag Therapeut. (2019). 16:139–43. 10.13929/j.1672-8475.201805029

[10] Lee CH Choi SW Jun SW Hwang J Kim IC Cho YK. Clinical impact of diabetes mellitus on 2-year clinical outcomes following PCI with second-generation drug-eluting stents; Landmark analysis findings from patient registry: pooled analysis of the Korean multicenter drug-eluting stent registry. PLoS ONE. (2020) 15:e0234362. 10.1371/journal.pone.0234362 32520973 PMC7286514

[11] Yokokawa T Yoshihisa A Kiko T Shimizu T Misaka T Yamaki T. Residual Gensini score is associated with long-term cardiac mortality in patients with heart failure after percutaneous coronary intervention. Circ Rep. (2020) 2:89–94. 10.1253/circrep.CR-19-0121 33693213 PMC7929761

[12] Reynolds HR Shaw LJ Min JK Page CB Berman DS Chaitman BR. Outcomes in the ISCHEMIA trial based on coronary artery disease and ischemia severity. Circulation. (2021) 144:1024–38. 10.1161/CIRCULATIONAHA.120.049755 34496632 PMC8478888

[13] Huo Y WYF. Training Materials for Interventional Therapy of Coronary Heart Disease, 2018 ed. Beijing: People's Medical Publishing House (2019). p. 103–6.

[14] Rampidis GP Benetos G Benz DC Giannopoulos AA Buechel RR. A guide for Gensini Score calculation. Atherosclerosis. (2019) 287:181–3. 10.1016/j.atherosclerosis.2019.05.012 31104809

[15] Farooq V van Klaveren D Steyerberg EW Meliga E Vergouwe Y Chieffo A. Anatomical and clinical characteristics to guide decision making between coronary artery bypass surgery and percutaneous coronary intervention for individual patients: development and validation of SYNTAX score II. Lancet. (2013) 381:639–50. 10.1016/S0140-6736(13)60108-7 23439103

[16] Matsumura-Nakano Y Shiomi H Morimoto T Yamaji K Ehara N Sakamoto H. Comparison of outcomes of percutaneous coronary intervention versus coronary artery bypass grafting among patients with three-vessel coronary artery disease in the new-generation drug-eluting stents era (from CREDO-Kyoto PCI/CABG registry cohort-3). Am J Cardiol. (2021) 145:25–36. 10.1016/j.amjcard.2020.12.076 33454340

[17] Wang H Wang H Wei Y Li X Jhummun V Ahmed MA. Ten-year outcomes of percutaneous coronary intervention versus coronary artery bypass grafting for patients with type 2 diabetes mellitus suffering from left main coronary disease: a meta-analysis. Diabetes Ther. (2021) 12:1041–54. 10.1007/s13300-021-01025-x 33641081 PMC7994472

[18] Wang J. The predictive value of SYNTAX score for the 1-year prognosis of patients undergoing percutaneous coronary intervention. Chin J Cardiovasc Dis. (2018) 46:267–73. 10.3760/cma.j.issn.0253-3758.2018.04.004 29747321

[19] Karayiannides S Norhammar A Frøbert O James SK Lagerqvist B Lundman P. Prognosis in patients with diabetes mellitus and STEMI undergoing primary PCI. J Am Coll Cardiol. (2018) 72:1427–8. 10.1016/j.jacc.2018.06.061 30213337

