# Supplementary material for: Predictive Value of Gensini Score in the Long-Term Outcomes of Patients With Coronary Artery Disease Who Underwent PCI
Source: Front Cardiovasc Med. 2022 Jan 24;8:778615. doi: 10.3389/fcvm.2021.778615 (PMC8818732; doi:10.3389/fcvm.2021.778615)
Supplement: Supplementary file 1 [file Table_1.docx]

**Supplementary-Table S1.Clinical outcomes and Gensini score according to tertiles**

| Clinical Outcomes | Gensini score | | | Χ^2^ | *P* value |
| --- | --- | --- | --- | --- | --- |
|  | ＜11 points | 11-38points | ＞38points |  |  |
| **CAD without diabetes（N=3562）** | **n=1251** | **n=1208** | **n=1103** |  |  |
| Stroke |  |  |  |  |  |
| N | 1236 (98.8) | 1193 (98.8) | 1089 (98.7) | 0.024 | 0.988 |
| Y | 15 (1.2) | 15 (1.2) | 14 (1.3) |  |  |
| Readmission |  |  |  |  |  |
| N | 1101 (88.0) | 1049 (86.8) | 943 (85.5) | 3.244 | 0.197 |
| Y | 150 (12.0) | 159 (13.2) | 160 (14.5) |  |  |
| Recurrent myocardial infarction |  |  |  |  |  |
| N | 1222 (97.7) | 1170 (96.9) | 1066 (96.6) | 2.550 | 0.279 |
| Y | 29 (2.3) | 38 (3.1) | 37 (3.4) |  |  |
| Target vessel revascularization. |  |  |  |  |  |
| N | 1203 (96.2) | 1150 (95.2) | 1039 (94.2) | 4.986 | 0.083 |
| Y | 48 (3.8) | 58 (4.8) | 64 (5.8) |  |  |
| Bleeding events |  |  |  |  |  |
| N | 1220 (97.5) | 1170 (96.9) | 1063 (96.4) | 2.651 | 0.266 |
| Y | 31 (2.5) | 38 (3.1) | 40 (3.6) |  |  |
| Heart failure |  |  |  |  |  |
| N | 1216 (97.2) | 1172 (97.0) | 1074 (97.4) | 0.261 | 0.878 |
| Y | 35 (2.8) | 36 (3.0) | 29 (2.6) |  |  |
| **CAD with diabetes（N=2110）** | **n=564** | **n=708** | **n=838** |  |  |
| Stroke |  |  |  |  |  |
| N | 557 (98.8) | 693 (97.9) | 828 (98.8) | 2.591 | 0.274 |
| Y | 7 (1.2) | 15 (2.1) | 10 (1.2) |  |  |
| Readmission |  |  |  |  |  |
| N | 498 (88.3) | 597 (84.3) | 720 (85.9) | 4.138 | 0.126 |
| Y | 66 (11.7) | 111 (15.7) | 118 (14.1) |  |  |
| Recurrent myocardial infarction |  |  |  |  |  |
| N | 544 (96.5) | 680 (96.0) | 812 (96.9) | 0.827 | 0.661 |
| Y | 20 (3.5) | 28 (4.0) | 26 (3.1) |  |  |
| Target vessel revascularization. |  |  |  |  |  |
| N | 539 (95.6) | 666 (94.1) | 782 (93.3) | 3.129 | 0.209 |
| Y | 25 (4.4) | 42 (5.9) | 56 (6.7) |  |  |
| Bleeding events |  |  |  |  |  |
| N | 552 (97.9) | 689 (97.3) | 819 (97.7) | 0.482 | 0.786 |
| Y | 12 (2.1) | 19 (2.7) | 19 (2.3) |  |  |
| Heart failure |  |  |  |  |  |
| N | 554 (98.2) | 683 (96.5) | 802 (95.7) | 6.689 | **0.035** |
| Y | 10 (1.8) | 25 (3.5) | 36 (4.3) |  |  |
