# Supplementary material for: Predictive Value of Gensini Score in the Long-Term Outcomes of Patients With Coronary Artery Disease Who Underwent PCI
Source: Front Cardiovasc Med. 2022 Jan 24;8:778615. doi: 10.3389/fcvm.2021.778615 (PMC8818732; doi:10.3389/fcvm.2021.778615)
Supplement: Supplementary file 2 [file Table_2.docx]

**Supplementary-Table S2. ACS between study groups**

|  | Total populatiom | |  |  |
| --- | --- | --- | --- | --- |
|  | Without DM | With DM | X^2^ | *P* value |
| ACS |  |  |  |  |
| N | 2134 (56.0) | 1164 (52.0) | 9.313 | 0.002 |
| Y | 1676 (44.0) | 1076 (48.0) |  |  |

**Abbreviations:** ACS: acute coronary syndrome, DM: diabetes mellitus
